# GITRL impairs hepatocyte repopulation by liver progenitor cells to aggravate inflammation and fibrosis by GITR^+^CD8^+^ T lymphocytes in CDE Mice

**DOI:** 10.1038/s41419-024-06506-y

**Published:** 2024-02-06

**Authors:** Li Li, Yu He, Kai Liu, Lin Liu, Shan Shan, Helin Liu, Jiangbo Ren, Shujie Sun, Min Wang, Jidong Jia, Ping Wang

**Affiliations:** 1 Liver Research Center, Beijing Friendship Hospital, Capital Medical University, Beijing, 100050 China; 2 National Clinical Research Center for Digestive Disease, Beijing, 100069 China; 3 Beijing Key Laboratory on Translational Medicine on Cirrhosis, Beijing, 100050 China; 4 Beijing Clinical Research Institute, Beijing, 100050 China

**Keywords:** Chronic inflammation, Stem-cell research

## Abstract

As an alternative pathway for liver regeneration, liver progenitor cells and their derived ductular reaction cells increase during the progression of many chronic liver diseases. However, the mechanism underlying their hepatocyte repopulation after liver injury remains unknown. Here, we conducted progenitor cell lineage tracing in mice and found that fewer than 2% of hepatocytes were derived from liver progenitor cells after 9 weeks of injury with a choline-deficient diet supplemented with ethionine (CDE), and this percentage increased approximately three-fold after 3 weeks of recovery. We also found that the proportion of liver progenitor cells double positive for the ligand of glucocorticoid-induced tumour necrosis factor receptor (GITRL, also called Tnfsf18) and SRY-related HMG box transcription 9 (Sox9) among nonparenchymal cells increased time-dependently upon CDE injury and reduced after recovery. When GITRL was conditionally knocked out from hepatic progenitor cells, its expression in nonparenchymal cells was downregulated by approximately fifty percent, and hepatocyte repopulation increased by approximately three folds. Simultaneously, conditional knockout of GITRL reduced the proportion of liver-infiltrating CD8^+^ T lymphocytes and glucocorticoid-induced tumour necrosis factor receptor (GITR)-positive CD8^+^ T lymphocytes. Mechanistically, GITRL stimulated cell proliferation but suppressed the differentiation of liver progenitor organoids into hepatocytes, and CD8^+^ T cells further reduced their hepatocyte differentiation by downregulating the Wnt/β-catenin pathway. Therefore, GITRL expressed by liver progenitor cells impairs hepatocyte differentiation, thus hindering progenitor cell-mediated liver regeneration.

## Introduction

Liver stem cells residing in the canal of Hering are transformed into proliferating progenitor cells that then differentiate into cholangiocytes or hepatocytes when liver damage occurs [1]. Due to the rare population of liver stem/progenitor cells in the normal liver and the enormous proliferation capacity of hepatocytes and cholangiocytes revealed by lineage-tracing, concerns have been raised about the existence of liver stem/progenitor cells and their contribution to liver regeneration [2, 3]. Recent single-cell RNA-sequencing studies have identified the liver stem/progenitor cell compartment both in the mouse liver [4] and in the liver of humans [5], which confirms their existence. However, although liver progenitor cells can differentiate into both cholangiocytes and hepatocytes in the liver of neonatal mice according to Prominin-1 (Prom1) lineage tracing, they only differentiate into cholangiocytes and lose hepatocyte differentiation potential after bile duct ligation [6]. Hepatocytes derived from liver progenitor cells are rare in physiological conditions [7]. They are also rarely observed after partial hepatectomy or the administration of carbon tetrachloride or 3,5-diethoxycarbonyl-1,4-dihydrocollidine-containing (DDC) diet in sex-determining region Y-Box 9 (Sox9) lineage-traced mice [7]. Even in a progenitor cell expansion model, less than 1% of hepatocytes are derived from Sox9- or osteopontin-expressing liver progenitor cells after 3 weeks of administering a choline-deficient chow supplemented with ethionine (CDE) [7, 8]. After 2 weeks of recovery from the CDE diet by readministering normal chow, osteopontin-marked liver progenitor cells contributed to 2.45% of the generated hepatocytes [8], which suggests that liver progenitor cell-derived hepatocytes more than doubled after liver injury. In addition, a 15-day CDE diet induced the generation of approximately 18% of cholangiocytes and 5% of hepatocytes from Forkhead Box L1 (Foxl1)-positive liver progenitor in the mice lost more than 14% of their initial body weight. After 4 days of recovery with normal chow, 50% of cholangiocytes and 29% of hepatocytes were derived from liver progenitor cells [9], suggesting that extensive injury promotes the differentiation of hepatocytes from liver progenitor cells. Since the number of liver progenitor cells increases during the progression of many human chronic liver diseases and is closely correlated with the extent of inflammation, fibrosis, and disease severity [10], it is critical to determine how to promote hepatocyte differentiation in the presence of liver injury, thus relieving inflammation and fibrosis.

Previous studies have reported some signalling molecules that mediate the expansion and/or differentiation of liver progenitor cells [11, 12]. The cytokines secreted by immune cells, such as interleukin-22 (IL-22) [13], tumour necrosis factor (TNF) [14], TNF-like weak inducer of apoptosis (TWEAK) [15], and lymphotoxin-β [16], can promote the expansion of liver progenitor cells. Wnt signalling [17–19] and hepatocyte growth factor [20] have been reported to play a role in the differentiation of liver progenitor cells into hepatocytes. Iloprost, a specific antagonist of connective tissue growth factor that blocks transforming growth factor (TGF)-β1, inhibits downstream fibrogenesis, and significantly increases the proportion of hepatocytes derived from liver progenitor cells [8], indicating that it may promote liver progenitor cell differentiation in vivo. Our previous study found that the specific ligand of glucocorticoid-induced tumour necrosis factor receptor (Tnfsf18, also called GITRL) for modulating immune cell activation is expressed by liver progenitor cells in human cirrhotic liver tissue and could be upregulated by TGF-β1 in liver progenitor cells to stimulate their proliferation in vitro [21]. Whether GITRL has any effects on liver progenitor cell differentiation into hepatocytes in vivo and whether specific depletion of GITRL in liver progenitor cells attenuates inflammation and fibrosis are the major questions to be answered.

In the present study, we found that GITRL is expressed by liver progenitor cells in vivo and that conditional knockout of GITRL from liver progenitor cells attenuates the bile ductular reaction and enhances hepatocyte differentiation in the presence of CDE injury. GITRL hinders the differentiation of liver progenitor cells into hepatocytes and reduces the recruitment of CD8^+^ T lymphocytes, and GITR further suppresses the differentiation of liver progenitor cells into hepatocytes by downregulating the Wnt/β-catenin pathway.

## Materials and methods

### Mice

Liver progenitor cell-specific lineage tracing mice were generated by breeding *Sox9*^*Cre-ER*^ mice (Shanghai Model Organisms, Shanghai, China) with *Rosa*^*TdTomato*^ mice (Shanghai Model Organisms) to generate *Sox9*^*Cre-ER*^*Rosa*^*TdTomato*^ mice. Genotyping was performed by PCR analysis of tail genomic DNA, and the male mice at 8 weeks of age were given one injection of 32 mg/kg body weight tamoxifen dissolved in corn oil by gavage [7]. After a 2-week washout period, the mice were given choline-deficient pelleted chow (Beijing Ke Ao Xie Li FEED Co., LTD, Beijing, China) and drinking water with 0.15% (wt/vol) ethionine [8] (J&K Scientific, Beijing China) as the CDE diet for 9 weeks (*N* = 3) and allowed to recover for 3 weeks with access to standard pelleted chow and water (*N* = 3).

For in vivo GITRL expression analysis, male C57BL/6 J mice at 8 weeks of age were purchased from Beijing HFK Bioscience Co. (Beijing, China) and maintained under specific pathogen-free conditions with free access to a CDE diet for 6 weeks (*N* = 6) or 9 weeks (*N* = 6). Nine-week CDE mice were allowed to recover from injury for 3 weeks and were provided standard pelleted chow and water during this time (*N* = 5).

The mice for specific conditional knockout of GITRL in liver progenitor cells were generated by breeding *Tnfsf18*^*+/flox*^ mice (Shanghai Model Organisms) with *Sox9*^*Cre-ER*^ transgenic mice and crossing their offspring to generate *Sox9*^*Cre-ER*^*Tnfsf18*^*flox/flox*^ mice and *Sox9*^*Cre-ER*^*Tnfsf18*^*+/+*^ mice. The male *Sox9*^*Cre-ER*^*Tnfsf18*^*flox/flox*^ mice and *Sox9*^*Cre-ER*^*Tnfsf18*^*+/+*^ mice at 8 weeks of age were given one injection of 32 mg/kg B.W. tamoxifen and fed standard pelleted chow and water for 2 weeks to wash out tamoxifen. Then, the *Sox9*^*Cre-ER*^*Tnfsf18*^*+/+*^ mice (*N* = 6) and *Sox9*^*Cre-ER*^*Tnfsf18*^*flox/flox*^ mice (*N* = 6) were fed a CDE diet for 9 weeks, and some other *Sox9*^*Cre-ER*^*Tnfsf18*^*+/+*^ mice (*N* = 6) and *Sox9*^*Cre-ER*^*Tnfsf18*^*flox/flox*^ mice (*N* = 6) were fed standard pelleted chow and water for 9 weeks as the uninjured control.

The mice for both GITRL knockout and liver progenitor cell tracing were generated by breeding *Sox9*^*Cre-ER*^*Tnfsf18*^*+/flox*^ mice with *Rosa*^*TdTomato*^ mice and crossing their offspring to generate *Sox9*^*Cre-ER*^*Rosa*^*TdTomato*^*Tnfsf18*^*flox/flox*^ mice and *Sox9*^*Cre-ER*^*Rosa*^*TdTomato*^*Tnfsf18*^*+/+*^ mice. The male *Sox9*^*Cre-ER*^*Rosa*^*TdTomato*^*Tnfsf18*^*+/+*^ mice (*N* = 6) and *Sox9*^*Cre-ER*^*Rosa*^*TdTomato*^*Tnfsf18*^*flox/flox*^ mice (*N* = 4) at 8 weeks of age were given one injection of 32 mg/kg B.W. tamoxifen and fed a CDE diet for 9 weeks after a 2-week washout of tamoxifen. As the uninjured control, *Sox9*^*Cre-ER*^*Rosa*^*TdTomato*^*Tnfsf18*^*+/+*^ mice (*N* = 4) and *Sox9*^*Cre-ER*^*Rosa*^*TdTomato*^*Tnfsf18*^*flox/flox*^ mice (*N* = 3) were given one injection of 32 mg/kg B.W. tamoxifen and fed standard pelleted chow and water for 9 weeks after 2-week washout of tamoxifen.

Mouse sample sizes were chosen based on previous similar experimental outcomes. The mice were assigned to experimental groups by random allocation. The investigator was blinded to the group allocation when assessing the outcome. All procedures were performed according to protocols approved by the Animal Care and Use Committee of Beijing Friendship Hospital, Capital Medical University (No. 19-2021).

### Double immunofluorescence

Paraffin-embedded liver tissue sections were used for standard immunofluorescence staining as described previously [22]. The sections were incubated with goat anti-TdTomato (1:400, Biorbyt, Catalogue Number: orb182397), mouse anti-cytokertin 19 (CK19) (1:200, RnD Systems, Catalogue Number: MAB3506), mouse anti-hepatocyte nuclear factor (HNF)4α (1:200, Abcam, Catalogue Number: ab41898), or rabbit anti-GITRL (1:150, ProteinTech, Catalogue Number: 23899-1-AP) primary antibodies. For visualization, the tissue sections were incubated with secondary antibodies conjugated to Alexa 488 (1:600; Molecular Probes, Catalogue Number: A-21202 or A-21206) and/or Alexa 555 (1:600; Molecular Probes, Catalogue Number: A-21432 or A-31572) under a Nikon 50i fluorescence microscope (Nikon, Japan) and scanned by a Panoramic MIDI Digital Slide Scanner (3DHISTECH, HUN).

### Flow cytometry analysis

Each mouse was perfused with 30 ml saline through the left ventriculus to remove circulating blood cells. Then, the liver tissue was removed and suspended in 10 ml Hanks’ Balanced Salt Solution (1×) with 0.1 mg/ml collagenase D and 0.01 mg/ml DNase I for further incubation at 37 °C for 30 min. After dissociation with a gentle‐MACS dissociator (Miltenyi Biotec, Bergisch‐Gladbach, Germany), the mixture was filtered through a 70‐μm nylon cell strainer and centrifuged at 50 g for 5 min. Then, the supernatant was centrifuged at 500 ×g for 20 min, and the cell pellet was resuspended in 5 ml 30% Percoll and centrifuged at 2000 rpm for 5 min twice. After washing with phosphate‐buffered saline (PBS), the nonparenchymal cells were incubated with mouse TruStain fcX^TM^ (Biolegend) to block nonspecific binding and divided into four groups. One group was immunostained with PE-labelled anti-CD45 (Biolegend, Catalogue Number: 147712), PE-Cy7-labelled anti-CD11b (Biolegend, Catalogue Number: 393104), BV510-labelled anti-GITRL (BD Bioscience, Catalogue Number: 563367) antibodies, and eFluor™ 780 (Thermo Fisher Scientific, Catalogue Number: 65-0865-14) for GITRL expression analysis in myeloid cells. The 2^nd^ group was immunostained with BV421-labelled anti-CD45 (Biolegend, Catalogue Number: 103134), PE-labelled anti-CD3 (Biolegend, Catalogue Number: 100206), PerCP-labelled anti-CD4 (Biolegend, Catalogue Number: 100434), APC/Cy7-labelled anti-CD8 (Biolegend, Catalogue Number: 100714), APC-labelled anti-glucocorticoid-induced tumour necrosis factor receptor (GITR, Biolegend, Catalogue Number: 126312) antibodies, and eFluor™ 780 (Thermo Fisher Scientific) for GITR expression analysis in T lymphocytes. The 3rd group was fixed, permeabilized and immunostained with BV421-labelled anti-CD45 (Biolegend, Catalogue Number: 103134), FITC-labelled anti-CD8 (Biolegend, Catalogue Number: 126606), PE-labelled anti-perforin (Biolegend, Catalogue Number: 154306), PerCP-labelled anti-granzyme B (Biolegend, Catalogue Number: 396416) antibodies, and eFluor™ 780 (Thermo Fisher Scientific) for CD8 function analysis. The last group was fixed, permeabilized and immunostained with PE-labelled anti-CD45 (Biolegend, Catalogue Number: 147712), FITC-labelled anti-EpCAM (Biolegend, Catalogue Number: 118208), Alexa Fluor 647-labelled anti-Sox9 (BD Biosciences, Catalogue Number: 565493), BV510-labelled anti-GITRL (Biolegend, Catalogue Number: 563367) antibodies, and eFluor™ 780 (Thermo Fisher Scientific) for GITRL expression analysis in liver progenitor cells. All samples were inputted into a FACS Aria II flow cytometer (BD Biosciences), and the data were analysed using FlowJo software (Treestar, Ashland, OR).

### Haematoxylin-eosin (HE) and Sirius red staining

Paraffin-embedded tissues were sectioned and stained with HE staining for routine pathologic examination or with Sirius red for visualization of extracellular matrix deposition. The HE- or Sirius red-stained sections were scanned by a Panoramic MIDI Digital Slide Scanner (3DHISTECH).

### RT‒PCR

Freshly isolated liver tissue was stored in RNA Stabilization Solution (Life Technologies, Carlsbad, CA) according to the manufacturer’s instructions. Total RNA extraction, reverse transcription, polymerase chain reaction, and data analysis were carried out according to methods described previously [23]. The primers are described in Supplementary Table 1, and the GAPDH gene was used as an endogenous reference.

### Organoid culture

Hepatic progenitor organoids were isolated by a tissue dissociation kit (CELLada Biotechnology, Beijing, China) according to the manufacturer’s instructions. In brief, liver tissue was mechanically dissected followed by dissociation for 30 min at 37 °C. The digested cells were filtered through a 100 μm cell strainer and washed with PBS at 1200 g and 4 °C for 5 min. Cells were suspended in DMEM/F12 medium, mixed with Matrigel Basement Membrane Matrix (Corning), and plated in a 24-well plate. After Matrigel polymerization, the cells were cultured with normal mouse liver tissue organoid culture medium (CELLada Biotechnology) at 37 °C and 5% CO_2_. The medium was changed every 2 days, and the cells were passaged after 6 days.

For GITR-Fc treatment, *Tnfsf18*^*+/+*^ organoids or *Tnfsf18*^*flox/flox*^ organoids were cultured with medium containing 2 μg/ml GITR-Fc or 2 μg/ml mouse immunoglobulin G (IgG) for 2 days.

For coculture of CD8^+^ T lymphocytes and liver progenitor organoids, CD8^+^ T lymphocytes were isolated by a mouse CD8^+^ T-cell isolation kit (Selleck) from mouse liver nonparenchymal cells according to the manufacturer’s instructions. *Tnfsf18*^*+/+*^ or *Tnfsf18*^*flox/flox*^ organoids were cultured with 1 × 10^6^ CD8^+^ T lymphocytes for 2 days.

### RNA sequencing and GSEA

RNA sequencing and library preparation of GITR-Fc-treated *Tnfsf18*^*+/+*^ organoids (*N* = 3) vs. IgG-treated *Tnfsf18*^*+/+*^ organoids (*N* = 3) or GITR-Fc-treated *Tnfsf18*^*flox/flox*^ organoids (*N* = 3) vs. IgG-treated *Tnfsf18*^*flox/flox*^ organoids (*N* = 3) were performed by Biomarker Biotechnology (Beijing, China). Sequencing data along with the study design have been submitted to the NCBI Sequence Read Archive and are available under study accession number PRJNA893027 (https://dataview.ncbi.nlm.nih.gov/object/PRJNA893027?reviewer=6b6mea30l1ritb6te51g631nq0). Differential expression analysis was performed using differential *P* values < 0.05 and log2(fold change) >1.0 or <−1.0. GSEA was performed by the R/clusterProfiler package and mouse gene sets were annotated using the Molecular Signatures Database [24]. A list of ranked genes from the RNA sequencing data of PRJNA893027 was used for GSEA performed by the R/Bioconductor package to compare the GITR-Fc-treated *Tnfsf18*^*+/+*^ vs. IgG-treated *Tnfsf18*^*+/+*^ organoids or GITR-Fc-treated *Tnfsf18*^*flox/flox*^ vs. IgG-treated *Tnfsf18*^*flox/flox*^ organoids.

### Statistics

All the experiments were repeated twice or at least had three biological repeats. The data were presented as the mean values ± SEM. The normal distributions of the data were analysed by SPSS on line (https://spssau.com/) and the data variances were estimated by GraphPad Prism 6 software (GraphPad, La Jolla, CA, USA). If the data variances were similar between groups, they were analysed for significance using an unpaired *T* test by two tailed *P* value with GraphPad Prism 6 software. If the data variances were not similar between groups, they were analysed for significance using an unpaired t test with Welch’s correction by two tailed *P* value with GraphPad Prism 6 software. *P* < 0.05 was considered a significant difference.

## Results

### The differentiation of liver progenitor cells into hepatocytes is inhibited upon CDE injury

**Fig. 1:**
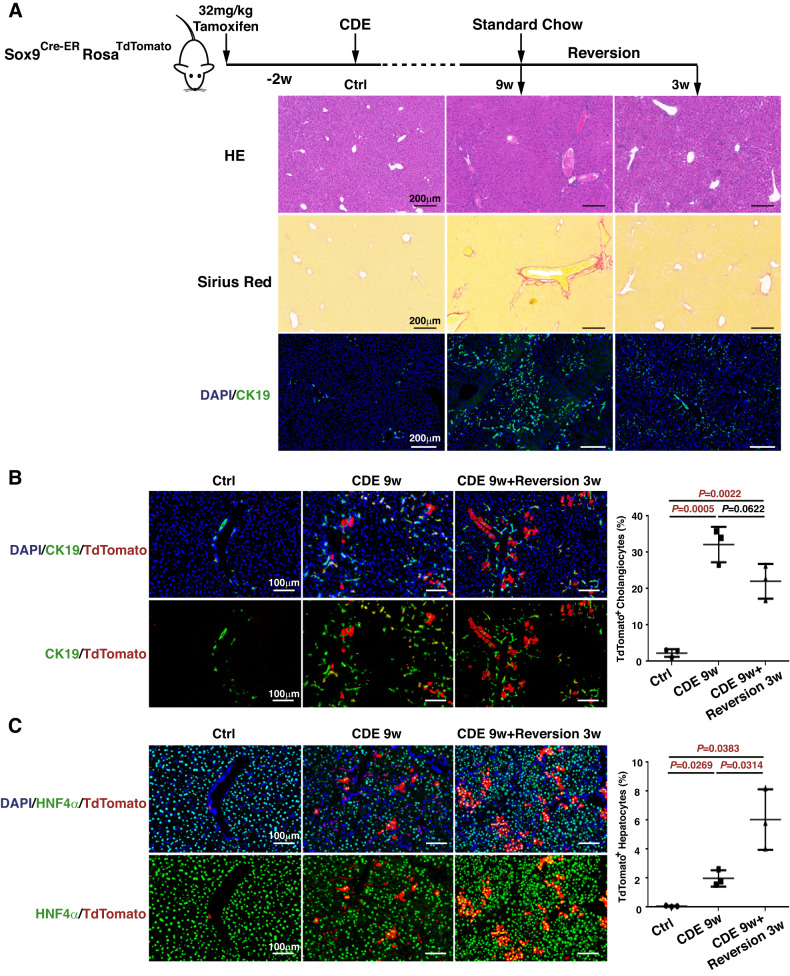
CDE liver injury inhibits the differentiation of liver progenitor cells into hepatocytes. **A** Tamoxifen-treated *Sox9*^*Cre-ER*^*Rosa*^*TdTomato*^ mice were exposed to CDE injury for 9 weeks and allowed to recover for 3 weeks (*N* = 3 per group). Liver sections were stained for HE (upper panel), Sirius red (middle panel), and CK19/DAPI (lower panel). **B** Representative immunofluorescence images and quantification of the percentage of TdTomato^+^CK19^+^ cells among CK19^+^ cells. **C** Representative immunofluorescence images and quantification of the percentage of TdTomato^+^HNF4α^+^ cells among HNF4α^+^ cells.

The CDE diet is a widely used regimen that activates liver progenitor cells by impairing hepatocyte function and transiently inhibiting hepatocyte proliferation [25]. Since a high dose of tamoxifen induces the ectopic expression of ductal markers in hepatocytes [26, 27], Sox9^+^ liver progenitor cells were specifically marked by a low dose of tamoxifen (32 mg/kg B.W.) and a 2-week washout period in *Sox9*^*Cre-ER*^*Rosa*^*TdTomato*^ mice [7]. Then, the mice were fed a CDE diet for 9 weeks and allowed to recover for 3 weeks on normal chow (Fig. 1A). Compared to the control mice, the 9-week CDE mice not only exhibited inflammatory cell infiltration as shown by HE staining (Fig. 1A) and extracellular matrix deposition as revealed by Sirius red staining (Fig. 1A) but also a severe ductular reaction in liver tissue as shown by CK19 immunostaining (Fig. 1A). After 3 weeks of recovery, liver inflammation, fibrosis, and the ductular reaction in the liver tissue were relieved (Fig. 1A). To reveal the fate of the Sox9^+^ liver progenitor cells, TdTomato and CK19 or HNF4α double immunostaining was conducted to determine their cholangiocyte or hepatocyte differentiation. In the uninjured control liver, TdTomato-labelled cells were strictly limited to CK19^+^ bile ducts with rare hepatocyte labelling (Fig. 1B, C). The 9-week CDE diet induced 32.14 ± 2.81% TdTomato^+^CK19^+^ cholangiocytes among CK19^+^ cholangiocytes, and this proportion was reduced to 22.04 ± 2.76% after 3 weeks of recovery, with no significant difference from that in 9-week CDE mice (Fig. 1B). Furthermore, the 9-week CDE diet resulted in only 1.99 ± 0.32% TdTomato^+^HNF4α^+^ hepatocytes among HNF4α^+^ hepatocytes, but this proportion increased to 6.04 ± 1.20% after 3 weeks of recovery, which was 3-fold more than that in 9-week CDE mice (Fig. 1C), indicating that CDE injury restricts the differentiation of liver progenitor cells into hepatocytes.

### The proportion of Sox9^+^GITRL^+^ liver progenitor cells increases upon CDE injury and decreases after recovery

**Fig. 2:**
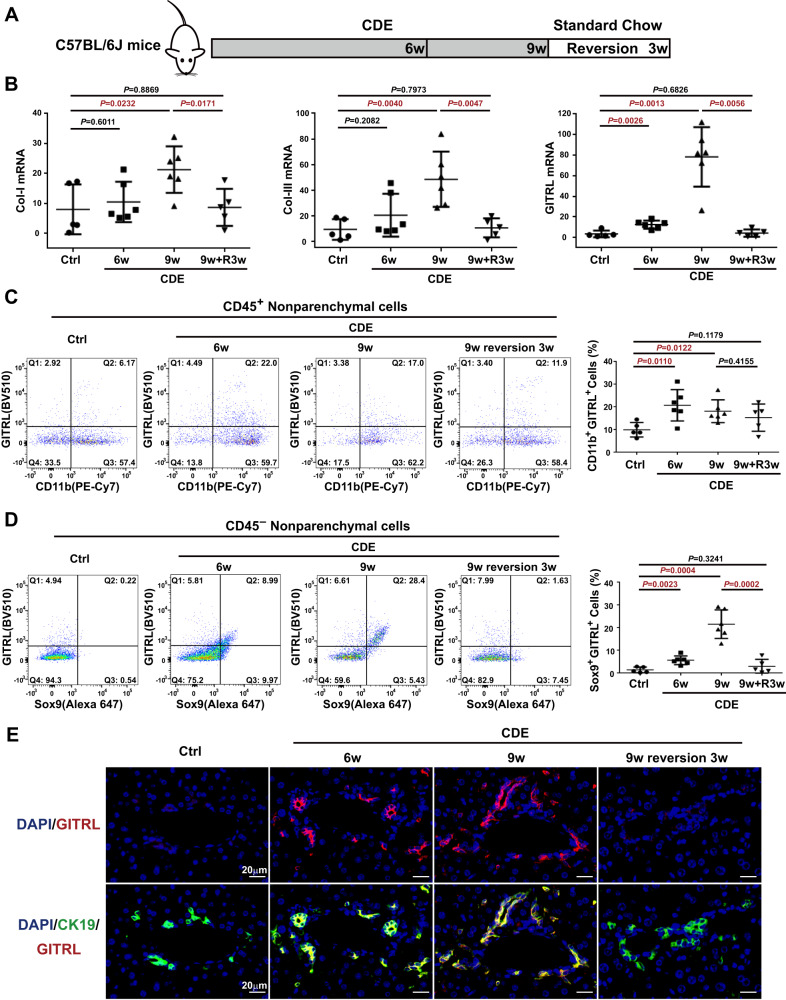
The proportion of Sox9^+^GITRL^+^ liver progenitor cells increased after CDE exposure and decreased after recovery. **A** Experimental design strategy of C57BL/6 mice exposed to CDE injury for 6 weeks or 9 weeks and allowed to recover for 3 weeks. **B** Tissue mRNA transcript levels of Collagen I, Collagen III, and GITRL at 6 weeks, 9 weeks after CDE exposure and 3 weeks of recovery. **C** Representative flow cytometry images and statistical quantification of the proportion of GITRL^+^CD11b^+^ myeloid cells among the liver-infiltrating CD45^+^ immune cells. **D** Representative flow cytometry images and statistical quantification of the proportion of Sox9^+^GITRL^+^ liver progenitor cells among CD45 ^–^ liver nonparenchymal cells. **E** Representative double immunofluorescence images of CK19, GITRL and DAPI.

Previously, we found that GITRL was the most significantly upregulated gene in rat liver progenitor cells after TGF-β1 incubation [21]. To confirm GITRL expression in the mouse liver tissue after CDE injury (Fig. 2A), real-time PCR data revealed the time-dependent increased transcript levels of collagen I, collagen III, and GITRL at 6 weeks and 9 weeks, which was reduced to the levels of the control mice after 3 weeks of recovery (Fig. 2B). To reveal GITRL-expressing cells, multicolour immune staining and flow cytometry analysis were used to analyse GITRL expression in liver-infiltrating myeloid cells and liver progenitor cells. First, neither the proportion of CD45^+^ immune cells nor the proportion of CD45^-^ nonparenchymal cells changed significantly after CDE injury and the recovery process (Fig. S1A, S1B). For GITRL expression in myeloid cells, the proportion of GITRL^+^CD11b^+^ myeloid cells in the CD45^+^ cells significantly increased to 20.77 ± 2.83% at 6 weeks (*P* = 0.0110) and 18.15 ± 2.06% at 9 weeks (*P* = 0.0122) after CDE injury compared to 9.99 ± 1.43% in the control mice (Fig. 2C). For GITRL expression in progenitor cells, the proportion of Sox9^+^GITRL^+^ liver progenitor cells in the CD45^-^ nonparenchymal cells also significantly increased to 5.65 ± 0.79% at 6 weeks (*P* = 0.0023) and 21.58 ± 2.58% at 9 weeks (*P* = 0.0004) after CDE injury compared to 1.41 ± 0.56% in the control mice (Fig. 2D). After 3 weeks of recovery, the proportion of GITRL^+^CD11b^+^ myeloid cells (15.31 ± 2.69%, *P* = 0.4155) was not markedly reduced (Fig. 2C), but the proportion of Sox9^+^GITRL^+^ cells (2.98 ± 1.38%, *P* = 0.0002) was significantly reduced compared to that at 9 weeks in CDE-fed mice (Fig. 2D). Similarly, the proportion of Sox9^+^EpCAM^+^ hepatic progenitor cells among CD45^-^ nonparenchymal cells showed a similar trend as Sox9^+^GITRL^+^ cells (Fig. S1C). In addition, double immunofluorescence of GITRL and CK19 revealed that CK19^+^ reactive bile ductules expressed GITRL in 6-week and 9-week CDE mouse livers (Fig. 2E), while the bile duct cells no longer expressed GITRL after 3 weeks of recovery (Fig. 2E). These data suggested that GITRL-positive liver progenitor cells dynamically change during injury and recovery and shows a reverse correlation with the differentiation of the lineage-traced liver progenitor cells into hepatocytes.

### Knocking out GITRL in liver progenitor cells attenuates ductular reaction and liver fibrosis

**Fig. 3:**
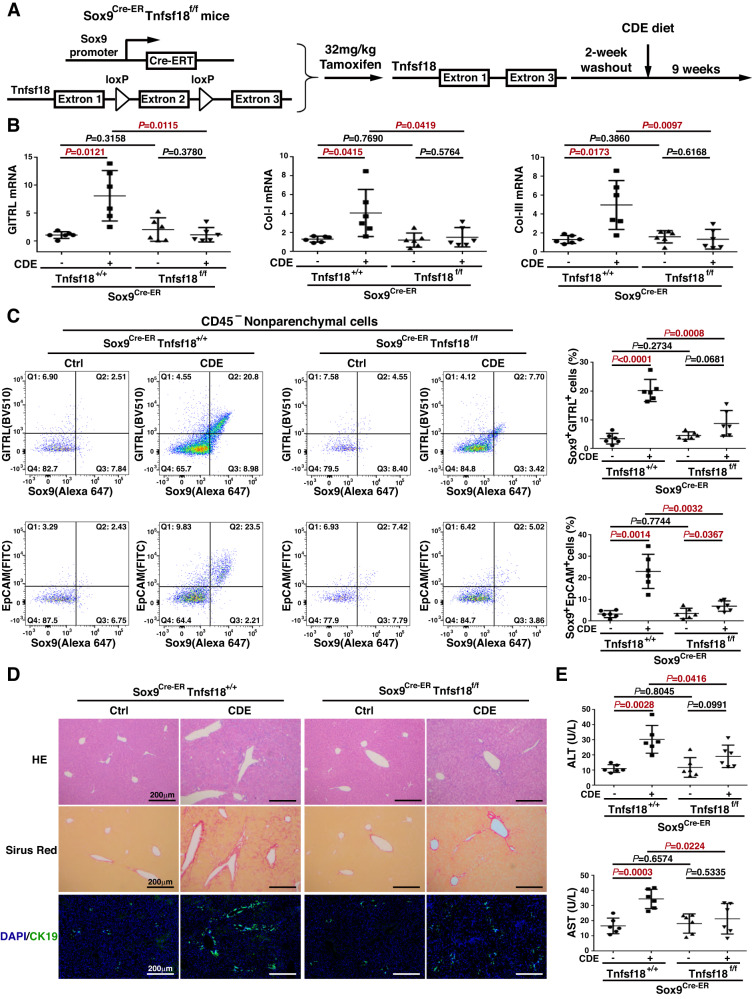
Knocking out GITRL in liver progenitor cells reduced liver inflammation, fibrosis and ductular reaction. **A** Experimental design strategy of *Sox9*^*Cre-ER*^*Tnfsf18*^*flox/flox*^ mice given a single dose of tamoxifen to inactivate GITRL followed by CDE injury after 2 weeks of tamoxifen washout. **B** Tissue mRNA transcript levels of Collagen I, Collagen III, and GITRL at 9 weeks in the CDE-treated *Sox9*^*Cre-ER*^*Tnfsf18*^*+/+*^ mice or *Sox9*^*Cre-ER*^*Tnfsf18*^*flox/flox*^ mice and their control mice. **C** Representative flow cytometry images and statistical quantifications of the proportion of GITRL^+^Sox9^+^ or Sox9^+^EpCAM^+^ liver progenitor cells among the CD45 ^–^ liver nonparenchymal cells at 9 weeks in the CDE-treated *Sox9*^*Cre-ER*^*Tnfsf18*^*+/+*^ mice or *Sox9*^*Cre-ER*^*Tnfsf18*^*flox/flox*^ mice and their control mice. **D** Liver sections were examined for HE (upper panel), Sirius red (middle panel), and CK19/DAPI (lower panel). **E** Serum ALT and AST levels of each group at 9 weeks after CDE exposure.

To reveal the effects of GITRL on liver progenitor cells in vivo, GITRL conditional knockout (*Sox9*^*Cre-ER*^*Tnfsf18*^*flox/flox*^) mice were generated by crossing the *Sox9*^*Cre-ER*^ line with a transgenic *Tnfsf18* locus line in which exon 2 is flanked by *loxP* sites. Following tamoxifen induction, Cre recombinase is expressed in Sox9-expressing liver progenitor cells, and *Tnfsf18* is inactivated (Fig. 3A). After 2 weeks of tamoxifen washout, liver injury was induced in *Sox9*^*Cre-ER*^*Tnfsf18*^*+/+*^ mice and *Sox9*^*Cre-ER*^*Tnfsf18*^*flox/flox*^ mice by a CDE diet for 9 weeks. The mRNA levels of GITRL, Collagen I, and Collagen III were significantly increased in the CDE-treated *Sox9*^*Cre-ER*^*Tnfsf18*^*+/+*^ mice compared to the control *Sox9*^*Cre-ER*^*Tnfsf18*^*+/+*^ mice but markedly reduced in CDE-treated *Sox9*^*Cre-ER*^*Tnfsf18*^*flox/flox*^ mice compared to CDE-treated *Sox9*^*Cre-ER*^*Tnfsf18*^*+/+*^ mice (Fig. 3B). Before analysing GITRL expression in myeloid cells and liver progenitor cells, we found that there was no significant change in the proportion of CD45^+^ immune cells or the proportion of CD45^-^ nonparenchymal cells in *Sox9*^*Cre-ER*^*Tnfsf18*^*+/+*^ mice or *Sox9*^*Cre-ER*^*Tnfsf18*^*flox/flox*^ mice in the presence or absence of CDE injury (Fig. S2A). The proportion of GITRL^+^CD11b^+^ myeloid cells in CD45^+^ liver infiltrating immune cells markedly increased in the CDE-treated *Sox9*^*Cre-ER*^*Tnfsf18*^*+/+*^ mice (*P* = 0.0014), yet conditional GITRL knockout did not significantly reduce this proportion (*P* = 0.3023, Fig. S2B). The proportion of GITRL^+^Sox9^+^ liver progenitor cells among CD45^-^ nonparenchymal cells was 20.23 ± 1.60% in the CDE-treated *Sox9*^*Cre-ER*^*Tnfsf18*^*+/+*^ mice and markedly reduced to 8.77 ± 1.82% in the CDE-treated *Sox9*^*Cre-ER*^*Tnfsf18*^*flox/flox*^ mice (*P* = 0.0008, Fig. 3C), which is approximately half depletion of GITRL^+^Sox9^+^ progenitor cells in GITRL-conditional knockout mice. Furthermore, the proportion of EpCAM^+^Sox9^+^ liver progenitor cells was also significantly reduced to 6.93 ± 0.98% in CDE-treated *Sox9*^*Cre-ER*^*Tnfsf18*^*flox/flox*^ mice compared to 23.05 ± 3.24% in CDE-treated *Sox9*^*Cre-ER*^*Tnfsf18*^*+/+*^ mice (*P* = 0.0032, Fig. 3C), which was also approximately 50% of the proportion in CDE-treated *Sox9*^*Cre-ER*^*Tnfsf18*^*+/+*^ mice. Simultaneously, GITRL conditional knockout reduced the immune cells around the portal area at 9 weeks after CDE injury compared to *Sox9*^*Cre-ER*^*Tnfsf18*^*+/+*^ mice by HE staining (Fig. 3D). Extracellular matrix deposition, as revealed by Sirius red staining (Fig. 3D), was also lower in CDE-treated *Sox9*^*Cre-ER*^*Tnfsf18*^*flox/flox*^ mice than in CDE-treated *Sox9*^*Cre-ER*^*Tnfsf18*^*+/+*^ mice. Furthermore, less reactive ductular were found in CDE-treated *Sox9*^*Cre-ER*^*Tnfsf18*^*flox/flox*^ mice than in CDE-treated *Sox9*^*Cre-ER*^*Tnfsf18*^*+/+*^ mice as assessed by CK19 immunostaining (Fig. 3D). The serum levels of alanine transaminase (ALT) and aspartate aminotransferase (AST), indicators of liver injury, were reduced in *Sox9*^*Cre-ER*^*Tnfsf18*^*flox/flox*^ mice compared to *Sox9*^*Cre-ER*^*Tnfsf18*^*+/+*^ mice (Fig. 3E). Therefore, conditional GITRL knockout in hepatic progenitor cells attenuates ductular reaction, liver inflammation, and liver fibrosis in CDE mice.

### GITRL depletion in liver progenitor cells reduces the proportions of liver-infiltrating CD8^+^ T lymphocytes and GITR^+^CD8^+^ T lymphocytes

To reveal the mechanism by which GITRL knockout affects liver fibrosis, the expression of GITR, the specific receptor for GITRL, was analysed in the human hepatic stellate cell line LX-II. LX-II cells did not express GITR based on flow cytometry results (Fig. S3A), and recombinant GITRL did not significantly upregulate their expression of collagen-I and collagen-III over 1.2-fold (Fig. S3B), suggesting that knocking out GITRL does not attenuate liver fibrosis by directly acting on hepatic stellate cells.

**Fig. 4:**
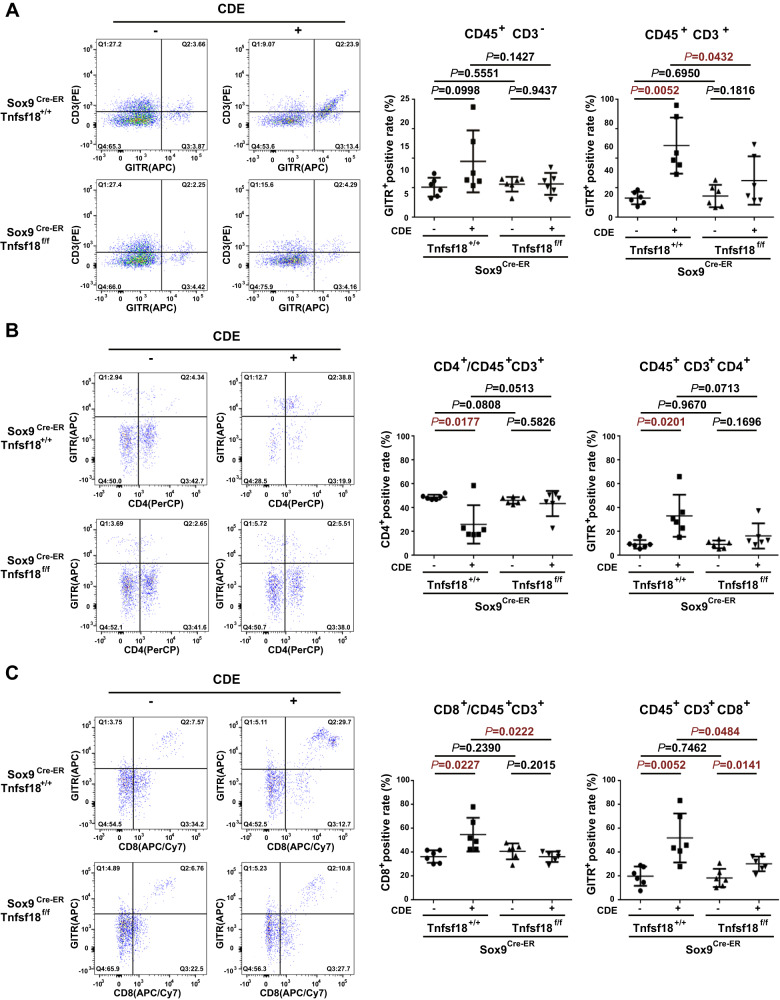
GITRL depletion in liver progenitor cells decreased the proportions of CD8^+^ lymphocytes and GITR^+^CD8^+^ lymphocytes. **A** Representative flow cytometry images and statistical quantification of the proportion of GITR^+^ cells among the liver-infiltrating CD45^+^CD3 ^–^ cells and CD45^+^CD3^+^ cells. **B** Representative flow cytometry images of the proportion of CD4^+^GITR^+^ cells among the liver-infiltrating CD45^+^CD3^+^ lymphocytes. Statistical quantification of the percentage of CD4^+^ cells among the CD45^+^CD3^+^ cells and GITR^+^ cells among CD45^+^CD3^+^CD4^+^ cells. **C** Representative flow cytometry images of the proportion of CD8^+^GITR^+^ cells among the liver-infiltrating CD45^+^CD3^+^ lymphocytes. Statistical quantification of the percentage of CD8^+^ cells among the CD45^+^CD3^+^ cells and GITR^+^ cells among CD45^+^CD3^+^CD8^+^ cells.

It has been reported that GITR is a marker of activated T lymphocytes and can costimulate T lymphocyte proliferation and activation after GITRL binding [28–30]. To reveal the immune cells involved in GITRL-associated liver progenitor cells, GITR-expressing cells after CDE injury were analysed. The uninjured *Sox9*^*Cre-ER*^*Tnfsf18*^*+/+*^ mice and *Sox9*^*Cre-ER*^*Tnfsf18*^*flox/flox*^ mice had similar GITR-positive proportions of both CD45^+^CD3 ^–^ immune cells and CD45^+^CD3^+^ T lymphocytes (Fig. 4A). After a 9-week CDE diet, the GITR-positive proportion did not change in CD45^+^CD3 ^–^ immune cells but was elevated in CD45^+^CD3^+^ T lymphocytes in *Sox9*^*Cre-ER*^*Tnfsf18*^*+/+*^ mice (Fig. 4A). However, there was no increased GITR-positive proportion of CD45^+^CD3^+^ T lymphocytes in *Sox9*^*Cre-ER*^*Tnfsf18*^*flox/flox*^ mice (Fig. 4A), indicating that knocking out GITRL in liver progenitor cells lowers the number of activated CD45^+^CD3^+^ T lymphocytes.

To identify the specific T lymphocyte subtype, the proportion of GITR-positive cells in CD4^+^ and CD8^+^ T lymphocytes was analysed. Nine weeks of CDE injury reduced the proportion of CD4^+^ T lymphocytes but enhanced the proportion of CD8^+^ T lymphocytes among CD45^+^CD3^+^ T lymphocytes in *Sox9*^*Cre-ER*^*Tnfsf18*^*+/+*^ mice, while there were no such changes in *Sox9*^*Cre-ER*^*Tnfsf18*^*flox/flox*^ mice (Fig. 4B, C). Nine weeks of CDE injury enhanced the proportion of GITR-positive cells in both CD4^+^ (Fig. 4B) and CD8^+^ T lymphocytes (Fig. 4C) from *Sox9*^*Cre-ER*^*Tnfsf18*^*+/+*^ mice, but GITRL conditional knockout mice only markedly reduced the proportion of GITR-positive cells in CD8^+^ T lymphocytes (Fig. 4C). However, functional analysis of CD8^+^ T lymphocytes showed that the expression of perforin (Fig. S2C) and granzyme B (Fig. S2D) did not change significantly in CDE-injured *Sox9*^*Cre-ER*^*Tnfsf18*^*flox/flox*^ mice compared to CDE-injured *Sox9*^*Cre-ER*^*Tnfsf18*^*+/+*^ mice. Since CD8^+^ T lymphocytes can aggravate liver fibrosis by secreting cytokines to activate hepatic stellate cells [31, 32], GITRL depletion in liver progenitor cells may attenuate liver fibrosis indirectly by reducing the total number of CD8^+^ T lymphocytes and their cytokine secretion.

### Loss of GITRL in liver progenitor cells enhances their differentiation into hepatocytes

**Fig. 5:**
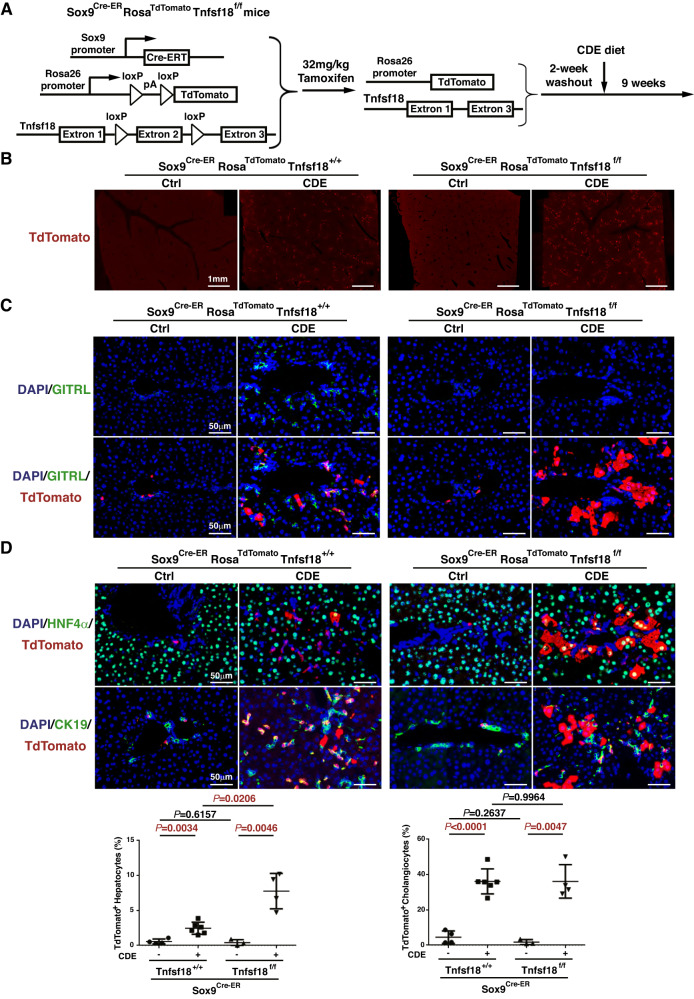
Knocking out GITRL in liver progenitor cells promoted hepatocyte repopulation. **A** Experimental design strategy of *Sox9*^*Cre-ER*^*Rosa*^*TdTomato*^*Tnfsf18*^*flox/flox*^ mice given a single dose of tamoxifen to inactivate GITRL and activate TdTomato followed by CDE injury after 2 weeks of tamoxifen washout. **B** Representative whole-slide scans of liver sections for TdTomato from the CDE-exposed *Sox9*^*Cre-ER*^*Rosa*^*TdTomato*^*Tnfsf18*^*+/+*^ mice or *Sox9*^*Cre-ER*^*Rosa*^*TdTomato*^*Tnfsf18*^*+/+*^ mice and their control mice. **C** Representative immunofluorescence images of TdTomato, GITRL and DAPI. **D** Representative immunofluorescence images of TdTomato, DAPI and CK19 or HNF4α. Statistical quantifications of the percentage of TdTomato^+^CK19^+^ cells among CK19^+^ cells or TdTomato^+^HNF4α^+^ cells among HNF4α^+^ cells.

To trace the fate of GITRL knockout liver progenitor cells, *Sox9*^*ER-Cre*^*Rosa*^*TdTomato*^*Tnfsf18*^*+/+*^ mice and *Sox9*^*ER-Cre*^*Rosa*^*TdTomato*^*Tnfsf18*^*flox/flox*^ mice were generated (Fig. 5A). Notably, there were more TdTomato^+^ areas in the liver tissue of *Sox9*^*ER-Cre*^*Rosa*^*TdTomato*^*Tnfsf18*^*flox/flox*^ mice than in *Sox9*^*ER-Cre*^*Rosa*^*TdTomato*^*Tnfsf18*^*+/+*^ mice after 9 weeks of CDE injury (Fig. 5B). Double immune staining of GITRL and TdTomato confirmed the depletion of GITRL in TdTomato^+^ liver progenitor cells in *Sox9*^*ER-Cre*^*Rosa*^*TdTomato*^*Tnfsf18*^*flox/flox*^ mice compared to those cells in *Sox9*^*ER-Cre*^*Rosa*^*TdTomato*^*Tnfsf18*^*+/+*^ mice (Fig. 5C). TdTomato-labelled cells were strictly limited to bile ducts with rare hepatocyte labelling in the control *Sox9*^*ER-Cre*^*Rosa*^*TdTomato*^*Tnfsf18*^*+/+*^ mice and control *Sox9*^*ER-Cre*^*Rosa*^*TdTomato*^*Tnfsf18*^*flox/flox*^ mice (Fig. 5D). Nine weeks of CDE injury induced the generation of 2.46 ± 0.35% TdTomato^+^HNF4α^+^ hepatocytes and 36.12 ± 2.89% TdTomato^+^CK19^+^ cholangiocytes in *Sox9*^*ER-Cre*^*Rosa*^*TdTomato*^*Tnfsf18*^*+/+*^ mice (Fig. 5D). When GITRL was knocked out in liver progenitor cells, the 9-week CDE diet induced the generation of 7.77 ± 1.27% TdTomato^+^HNF4α^+^ hepatocytes and 36.15 ± 4.75% TdTomato^+^CK19^+^ cholangiocytes in *Sox9*^*ER-Cre*^*Rosa*^*TdTomato*^*Tnfsf18*^*flox/flox*^ mice. The number of liver progenitor-derived cholangiocytes was similar but *Sox9*^*ER-Cre*^*Rosa*^*TdTomato*^*Tnfsf18*^*flox/flox*^ mice exhibited a 3-fold increase in the number of liver progenitor-derived hepatocytes compared to that in *Sox9*^*ER-Cre*^*Rosa*^*TdTomato*^*Tnfsf18*^*+/+*^ mice (Fig. 5D), suggesting that depletion of GITRL in liver progenitor cells increases progenitor cell differentiation into hepatocytes in the presence of injury.

### GITRL impairs the differentiation of liver progenitor cells into hepatocytes, and GITR further inhibits this process by downregulating the Wnt/β-catenin pathway

**Fig. 6:**
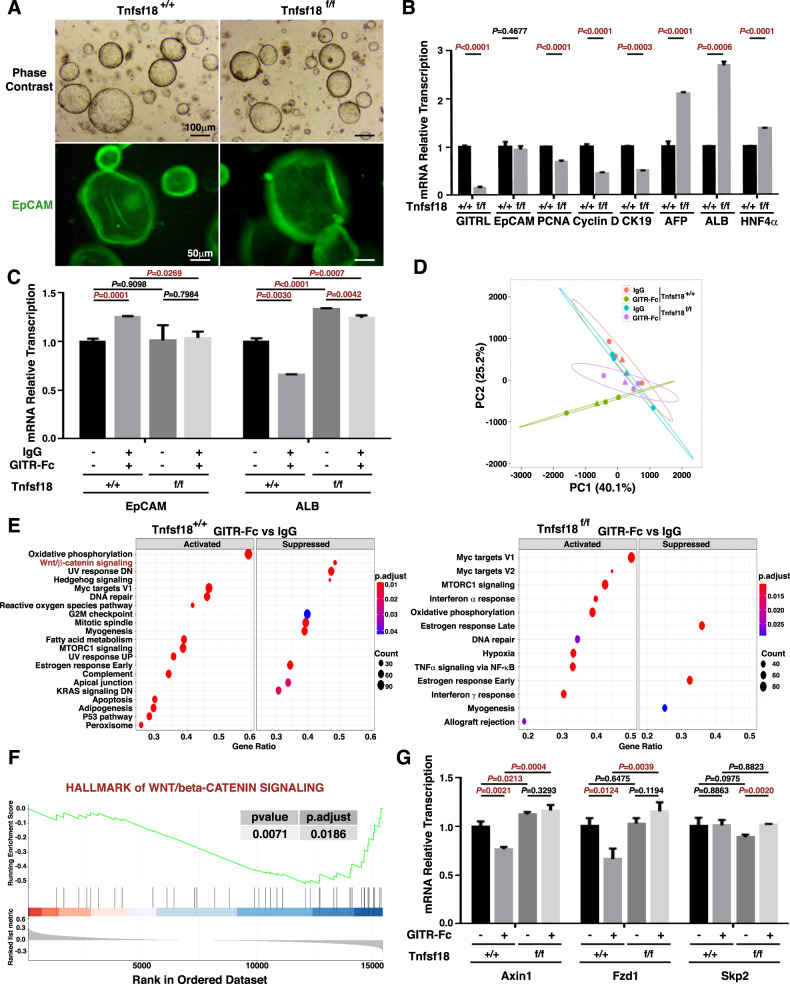
GITRL suppresses the differentiation of liver progenitor cells into hepatocytes, and GITR further hinders this process by reducing the Wnt/β-catenin pathway. **A** Representative bright-field microscopy images or EpCAM immunofluorescence images of the liver progenitor organoids from *Sox9*^*Cre-ER*^*Rosa*^*TdTomato*^*Tnfsf18*^*+/+*^ mice or *Sox9*^*Cre-ER*^*Rosa*^*TdTomato*^*Tnfsf18*^*flox/flox*^ mice. **B** The mRNA transcript levels of GITRL, EpCAM, PCNA, Cyclin D, CK19, AFP, and ALB in the *Sox9*^*Cre-ER*^*Rosa*^*TdTomato*^*Tnfsf18*^*+/+*^ or *Sox9*^*Cre-ER*^*Rosa*^*TdTomato*^*Tnfsf18*^*flox/flox*^ liver progenitor organoids. **C** The mRNA transcript levels of EpCAM and ALB in the GITR-Fc-exposed *Sox9*^*Cre-ER*^*Rosa*^*TdTomato*^*Tnfsf18*^*+/+*^ or *Sox9*^*Cre-ER*^*Rosa*^*TdTomato*^*Tnfsf18*^*flox/flox*^ liver progenitor organoids. **D** Principal component analysis (PCA) of GITR-Fc- or IgG-exposed *Sox9*^*Cre-ER*^*Rosa*^*TdTomato*^*Tnfsf18*^*+/+*^ or *Sox9*^*Cre-ER*^*Rosa*^*TdTomato*^*Tnfsf18*^*flox/flox*^ liver progenitor organoids. **E** GSEA of GITR-Fc-exposed *Sox9*^*Cre-ER*^*Rosa*^*TdTomato*^*Tnfsf18*^*+/+*^ liver progenitor organoids v.s. their IgG-exposed organoids and GITR-Fc-exposed *Sox9*^*Cre-ER*^*Rosa*^*TdTomato*^*Tnfsf18*^*flox/flox*^ liver progenitor organoids v.s. their IgG-exposed organoids. **F** GSEA revealed suppression of Wnt/β-catenin signalling in GITR-Fc-exposed *Sox9*^*Cre-ER*^*Rosa*^*TdTomato*^*Tnfsf18*^*+/+*^ liver progenitor organoids v.s. their IgG-exposed organoids. **G** The mRNA transcript levels of Axin1, Fzd1, and Skp2 in the GITR-Fc-exposed *Sox9*^*Cre-ER*^*Rosa*^*TdTomato*^*Tnfsf18*^*+/+*^ or *Sox9*^*Cre-ER*^*Rosa*^*TdTomato*^*Tnfsf18*^*flox/flox*^ liver progenitor organoids and their IgG-exposed control organoids.

To gain insights into the mechanism by which GITRL depletion enhances hepatocyte differentiation, liver progenitor cells were isolated from the livers of *Sox9*^*Cre-ER*^*Rosa*^*TdTomato*^*Tnfsf18*^*+/+*^ mice and *Sox9*^*Cre-ER*^*Rosa*^*TdTomato*^*Tnfsf18*^*flox/flox*^ mice by collagenase digestion and cultured as 3-dimensional (3D) organoids (Fig. 6A). Both of the organoids consutrcted from *Sox9*^*Cre-ER*^*Rosa*^*TdTomato*^*Tnfsf18*^*+/+*^ mice and *Sox9*^*Cre-ER*^*Rosa*^*TdTomato*^*Tnfsf18*^*flox/flox*^ mice were positive for EpCAM (Fig. 6A), indicating their progenitor phenotype. Real-time PCR analysis showed that GITRL mRNA levels in *Sox9*^*Cre-ER*^*Rosa*^*TdTomato*^*Tnfsf18*^*flox/flox*^ organoids were only one tenth of those in *Sox9*^*Cre-ER*^*Rosa*^*TdTomato*^*Tnfsf18*^*+/+*^ organoids (Fig. 6B), and GITRL knockout significantly suppressed the transcription of proliferating cell nuclear antigen (PCNA), cyclin D, and CK19 while markedly enhancing the transcription of albumin (ALB), α-fetoprotein (AFP) and HNF4α(Fig. 6B), suggesting that GITRL enhances cell proliferation and represses the hepatocyte phenotype.

Since GITRL has a cytoplasmic domain, it can transduce a receptor‒ligand reverse signal after binding with its specific receptor, GITR [33]. After 2 days of GITR-Fc or IgG incubation, there was increased transcription of EpCAM and reduced transcription of ALB in *Sox9*^*Cre-ER*^*Rosa*^*TdTomato*^*Tnfsf18*^*+/+*^ organoids (Fig. 6C), but there were no such significant changes in *Sox9*^*Cre-ER*^*Rosa*^*TdTomato*^*Tnfsf18*^*flox/flox*^ organoids (Fig. 6C). Then, RNA sequencing and principal component analysis (PCA) were conducted, and the PCA showed that GITR-Fc treatment resulted in a great difference between the two kinds of organoids, and the GITR-Fc-treated *Sox9*^*Cre-ER*^*Rosa*^*TdTomato*^*Tnfsf18*^*flox/flox*^ organoids were closer to the untreated organoids than the GITR-Fc-treated *Sox9*^*Cre-ER*^*Rosa*^*TdTomato*^*Tnfsf18*^*+/+*^ organoids (Fig. 6D). Further GSEA showed that Wnt/β-catenin signalling ranked as the most suppressed pathway in *Sox9*^*Cre-ER*^*Rosa*^*TdTomato*^*Tnfsf18*^*+/+*^ organoids, while it was not suppressed in *Sox9*^*Cre-ER*^*Rosa*^*TdTomato*^*Tnfsf18*^*flox/flox*^ organoids (Fig. 6E, F). Among the genes of the Wnt/β-catenin pathway, the transcription of S-Phase Kinase-Associated Protein 2 (Skp2) did not change much, while Axis Inhibition Protein 1 (Axin1) and Frizzled Family Receptor 1 (Fzd1) were markedly downregulated after GITR-Fc incubation in *Sox9*^*Cre-ER*^*Rosa*^*TdTomato*^*Tnfsf18*^*+/+*^ organoids; there were no such changes in *Sox9*^*Cre-ER*^*Rosa*^*TdTomato*^*Tnfsf18*^*flox/flox*^ organoids (Fig. 6G), confirming the suppression of the Wnt/β-catenin pathway after GITR/GITRL interaction. The Wnt/β-catenin pathway is involved in the differentiation of liver progenitor cells into hepatocytes [17–19], and GITR inhibits the differentiation of liver progenitor cells into hepatocytes by suppressing this pathway.

### GITR and GITRL are involved in the interaction between CD8^+^ T lymphocytes and liver progenitor organoids

**Fig. 7:**
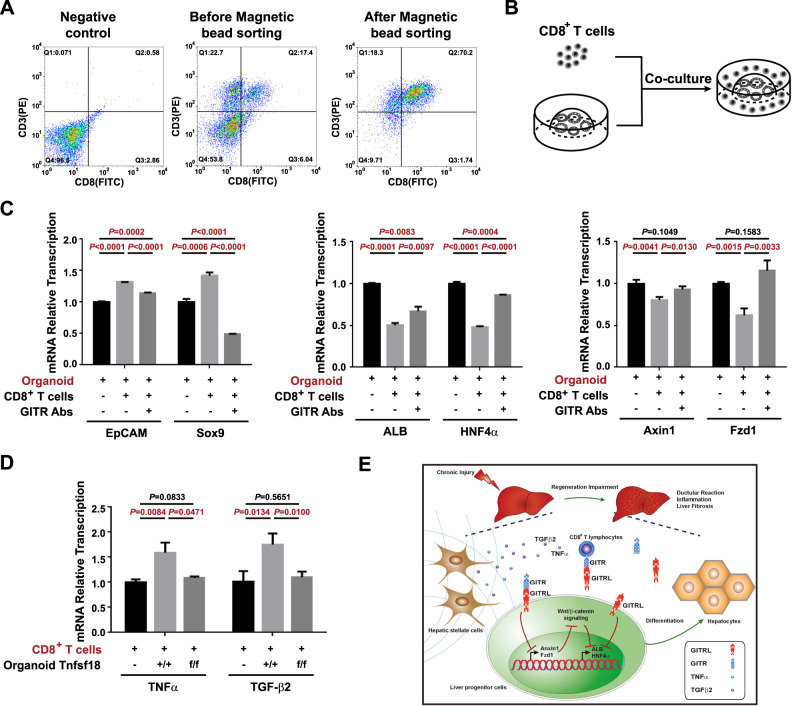
GITR and GITRL participate in the interaction of CD8^+^ T lymphocytes and liver progenitor organoids. **A** Representative flow cytometry images of the proportion of CD8^+^ T lymphocytes before and after magnetic sorting from the nonparenchymal cells. **B** Experimental design strategy of the coculture system of CD8^+^ T lymphocytes and liver progenitor organoids. **C** The mRNA transcript levels of EpCAM, Sox9, ALB, HNF4α, Axin1, and Fzd1 in liver progenitor organoids cocultured with CD8^+^ T lymphocytes with or without GITR antibodies. **D** The mRNA transcript levels of TNFα and TGF-β2 in CD8^+^ T lymphocytes cocultured with *Sox9*^*Cre-ER*^*Rosa*^*TdTomato*^*Tnfsf18*^*+/+*^ or *Sox9*^*Cre-ER*^*Rosa*^*TdTomato*^*Tnfsf18*^*flox/flox*^ liver progenitor organoids. **E** Schematic representation of the effect of GITR and GITRL on hepatic progenitor cells and CD8^+^ T lymphocytes in CDE mice.

Considering that GITRL knockout in liver progenitor cells mainly affects CD8^+^ T lymphocytes and GITR^+^CD8^+^ T lymphocytes as revealed by previous mouse experiments, a coculture system of 3D progenitor organoids and magnetic bead-sorted CD8^+^ T lymphocytes was designed to study their interaction (Fig. 7A, B). CD8^+^ T lymphocytes significantly increased the transcription of EpCAM and Sox9 and markedly suppressed the transcription of ALB, HNF4α, Axin1, and Fzd1 in *Sox9*^*Cre-ER*^*Rosa*^*TdTomato*^*Tnfsf18*^*+/+*^ organoids (Fig. 7C), while GITR antibodies blocked these effects (Fig. 7C), suggesting that CD8^+^ T lymphocytes inhibit the differentiation of liver progenitor cells into hepatocytes via the GITR and Wnt/β-catenin pathway. Moreover, *Sox9*^*Cre-ER*^*Rosa*^*TdTomato*^*Tnfsf18*^*+/+*^ organoids markedly stimulated the transcription of TNFα and TGF-β2 in CD8^+^ T lymphocytes (Fig. 7D), while *Sox9*^*Cre-ER*^*Rosa*^*TdTomato*^*Tnfsf18*^*flox/flox*^ organoids showed no such effects (Fig. 7D), indicating that liver progenitor organoids promote inflammatory and fibrogenetic cytokines expression of CD8^+^ T lymphocytes by mediating GITRL. Therefore, GITR and GITRL signalling suppresses the differentiation of liver progenitor cells into hepatocytes and enhances the inflammation- and fibrosis-stimulating effects of CD8^+^ T lymphocytes (Fig. 7E).

## Discussion

The number of reactive bile ductular cells, which are mainly derived from liver progenitor cells, is correlated with the severity of chronic liver diseases. Inducing the differentiation of liver progenitor cells into hepatocytes may serve as a key strategy to improve the clinical outcome of chronic liver diseases. This study provides three new findings that improve our understanding of the pathological mechanisms underlying liver progenitor cell differentiation into hepatocytes. First, GITRL is responsible for suppressing the differentiation of liver progenitor cells into hepatocytes. Second, GITR further inhibits the differentiation of liver progenitor cells into hepatocytes by suppressing the Wnt/β-catenin pathway. Last, depletion of GITRL in liver progenitor cells attenuates liver inflammation and fibrosis by reducing the recruitment of CD8^+^ T lymphocytes and cytokine release, thus providing a new therapeutic strategy for relieving liver injury.

It is well known that GITRL is expressed by antigen-presenting cells and plays an irreplaceable role in inflammation and fibrosis [34]. In this study, we found that CDE injury increased the proportion of GITRL^+^CD11b^+^ myeloid cells, but conditional knockout of GITRL in liver progenitor cells did not markedly change the proportion of GITRL^+^CD11b^+^ myeloid cells in CDE-injured *Sox9*^*Cre-ER*^*Tnfsf18*^*flox/flox*^ mice compared to CDE-injured *Sox9*^*Cre-ER*^*Tnfsf18*^*+/+*^ mice. Therefore, the effects of lowering the ductular reaction, reducing CD8^+^ T lymphocytes, and attenuating fibrosis are the results of knocking down GITRL in liver progenitor cells, not by GITRL-expressing myeloid cells. Furthermore, we found that knocking out GITRL markedly reduces the proportion of liver progenitor cells and induces the generation of HNF4α^+^ hepatocytes (7.77%) by 3-fold in the presence of CDE injury compared to that (2.46%) in GITRL normal mice. Similarly, the administration of iloprost resulted in the generation of 3.26% HNF4α^+^ hepatocytes derived from osteopontin lineage-traced liver progenitor cells compared to 0.65% in PBS-treated mice after 3 weeks of CDE injury [8], which promoted the differentiation of liver progenitor cells into hepatocytes 5-fold by reducing laminin levels. Mechanistically, we found that GITRL could increase the mRNA levels of PCNA and cyclin D, which are involved in cell proliferation, and decrease the mRNA levels of AFP, ALB, and HNF4α, which control the hepatocyte phenotype. Therefore, GITRL is the molecule expressed by liver progenitor cells that generates proliferating bile ductular cells and suppresses hepatocyte differentiation.

In addition to being a ligand, GITRL can transduce intercellular signals when bound to GITR via its cytoplasmic domain [33]. In the presence of GITR-Fc, GITRL further reduces the hepatocyte phenotype by suppressing the Wnt/β-catenin pathway, which is involved in hepatocyte differentiation [17–19]. CD8^+^ T lymphocytes could suppress hepatocyte phenotype gene expression (ALB and HNF4α) and Wnt/β-catenin pathway gene expression (Axin1 and Fzd1) in liver progenitor organoids, while GITR antibodies abrogated these effects, showing that CD8^+^ T lymphocytes inhibited the differentiation of liver progenitor cells into hepatocytes via GITR and the Wnt/β-catenin pathway.

It has been reported that cholangiocytes can serve as antigen-presenting cells to activate liver-infiltrating natural killer T cells [35] and mucosal-associated invariant T cells [36]. We found that conditional depletion of GITRL in liver progenitor cells markedly reduced the proportions of liver-infiltrating CD8^+^ T lymphocytes and GITR^+^CD8^+^ T lymphocytes, supporting GITRL as a molecule that impacts CD8^+^ T lymphocytes. Although the expression of perforin and granzyme B is not affected by GITRL on liver progenitor cells in vivo, in vitro coculture data revealed that GITRL expressed by liver progenitor organoids could stimulate the expression of the inflammatory cytokine TNFα and the fibrogenic cytokine TGF-β2, which is consistent with previous studies on biliary epithelial cells to activate immune cells [35, 36].

In summary, GITRL is a molecule expressed by liver progenitor cells that recruits CD8^+^ T lymphocytes, which can stimulate cell proliferation but impair the differentiation of liver progenitor cells into hepatocytes. Abrogation of GITRL in liver progenitor cells could alleviate ductular reactions, liver inflammation, and liver fibrosis, thus serving as a new target for promoting liver regeneration.

### Supplementary information

Author contribution

Supplementary table 1

Supplementary figures

English Editing Certificate.

## Data Availability

RNA sequencing data along with the study design are available under study accession number PRJNA893027 in accordance with funder data-retention policies.
