# Supplementary material for: GITRL impairs hepatocyte repopulation by liver progenitor cells to aggravate inflammation and fibrosis by GITR+CD8+ T lymphocytes in CDE Mice
Source: Cell Death Dis. 2024 Feb 6;15(2):114. doi: 10.1038/s41419-024-06506-y (PMC10847460; doi:10.1038/s41419-024-06506-y)
Supplement: Supplementary file 1 — Author contribution [file 41419_2024_6506_MOESM1_ESM.pdf]

DECLARATION OF CONTRIBUTIONS TO ARTICLE **ADMC**

Manuscript Number:	Journal Name:	
CDDIS-23-0032RRR	Cell Death & Disease	(the 'Journal')

Proposed Title of the Contribution:

GITRL Impairs Hepatocyte Repopulation by Liver Progenitor Cells to Aggravate Inflammation and Fibrosis by GITR+CD8+ T Lymphocytes in CDE Mice. (the 'Contribution')

Author(s):

Li Li, Yu He, Kai Liu, Lin Liu, Shan Shan, Helin Liu, Jiangbo Ren, Shujie Sun, Min Wang, Jidong Jia, and Ping Wang. (the 'Authors')

For all *CDD* articles, each person named as an author in the published version must be able to show he or she has contributed substantially to the article.

Authorship credit should be based on 1) substantial contributions to conception and design, acquisition of data, or analysis and interpretation of data; 2) drafting the article or revising it critically for important intellectual content; and 3) final approval of the version to be published. Authors should meet conditions 1, 2 and 3.

Any person who cannot be shown to have made a substantial contribution to the article cannot be listed as an author in the final version. The name of any person who is deemed to have made a minor contribution can, however, appear in the Acknowledgments section of the article.

Please complete the table below to indicate the contributions of all named authors to the manuscript.

Author Full Name:	Specification of Contribution to the Manuscript:
Li Li	Acquisition and analysis of the data.
Yu He	Acquisition of the data.
Kai Liu	Technical support and helping acquisition of data.
Lin Liu	Material support and helping acquisition of the data.
Shan Shan	Material support and helping acquisition of the data.
Helin Liu	Technical support and helping analysing the data.
Jiangbo Ren	Technical support and helping acquisition of data.
Shujie Sun	Technical support and helping acquisition of data.
Min Wang	Technical support and helping acquisition of data.
Jidong Jia	Conception and design, interpretation of data.
Ping Wang	Conception and design,acquisition and analysis of the data.

Please complete the table below to indicate the contributions of all named authors to the figures.

Figure 1:

Li Li, Yu He, and Kai Liu generated the data.
Li Li, Helin Liu, and Ping Wang analysed the data and prepared the figure.

Figure 2:

Li Li and Lin Liu generated the data for panel B.
Li Li, Yu He, and Renjiangbo generated the data for panel C and D.
Li Li, Yu He, and Kai Liu generated the data for panel E.
Li Li, Helin Liu, and Ping Wang analysed the data and prepared the figure.

Figure 3:

Li Li and Lin Liu generated the data for panel B.
Li Li, Yu He, and Renjiang Bo generated the data for panel C.
Li Li, Yu He, and Kai Liu generated the data for panel D.
Li Li, Yu He, and Shujie Sun generated the data for panel E.
Li Li, Helin Liu, and Ping Wang analysed the data and prepared the figure.

Figure 4:

Li Li, Yu He, and Jiangbo Ren generated the data.
Li Li, Helin Liu, and Ping Wang analysed the data and prepared the figure.

Figure 5:

Yu He, Li Li, and Kai Liu generated the data.
Li Li, Helin Liu, and Ping Wang analysed the data and prepared the figure.

Figure 6:

Li Li, Shan Shan, Min Wang, and Ping Wang generated the data.
Li Li, Shan Shan, and Ping Wang analysed the data and prepared the figure.

Figure 7:

Li Li, Shan Shan, Min Wang, and Ping Wang generated the data.
Li Li, Shan Shan, and Ping Wang analysed the data and prepared the figure.

Signed for and on behalf of the Author(s):	Print Name:	Date:
Ping Wang	Ping Wang	1/25/24
