# Supplementary material for: GITRL impairs hepatocyte repopulation by liver progenitor cells to aggravate inflammation and fibrosis by GITR+CD8+ T lymphocytes in CDE Mice
Source: Cell Death Dis. 2024 Feb 6;15(2):114. doi: 10.1038/s41419-024-06506-y (PMC10847460; doi:10.1038/s41419-024-06506-y)
Supplement: Supplementary file 2 — Supplementary table 1 [file 41419_2024_6506_MOESM2_ESM.docx]

Table S1. Mouse primers for RT-PCR analysis.

| Name | Primer sequence | Gene symbol |
| --- | --- | --- |
| AFP | F: CTTCCCTCATCCTCCTGCTAC | NM_007423.4 |
|  | R: ACAAACTGGGTAAAGGTGATGG |  |
| Alb | F: TGCTTTTTCCAGGGGTGTGTT | NM_009654.4 |
|  | R: TTACTTCCTGCACTAATTTGGCA |  |
| Axin1 | F: CTCCAAGCAGAGGACAAAATCA  R: GGATGGGTTCCCCACAGAAATA | NM_001159598.2 |
| CK19 | F: GGGGGTTCAGTACGCATTGG | NM_008471.3 |
|  | R: GAGGACGAGGTCACGAAGC |  |
| Col-I | F: GCTCCTCTTAGGGGCCACT | NM_007742.4 |
|  | R: CCACGTCTCACCATTGGGG |  |
| Col-III | F: CTGTAACATGGAAACTGGGGAAA | NM_009930.2 |
|  | R: CCATAGCTGAACTGAAAACCACC |  |
| Cyclin D | F: GCGTACCCTGACACCAATCTC | NM_001379248.1 |
|  | R: CTCCTCTTCGCACTTCTGCTC |  |
| EpCAM | F: GCGGCTCAGAGAGACTGTG | NM_008532.2 |
|  | R: CCAAGCATTTAGACGCCAGTTT |  |
| Fzd1 | F: CAGCAGTACAACGGCGAAC | NM_021457.3 |
|  | R: GTCCTCCTGATTCGTGTGGC |  |
| GAPDH | F: GGAGCGAGATCCCTCCAAAAT | NM_002046.7 |
|  | R: GGCTGTTGTCATACTTCTCATGG |  |
| GITRL | F: ATGGAGGAAATGCCTTTGAGAG | NM_183391.4 |
|  | R: GATGGCAGTTGGCTTGAGTGA |  |
| HNF4α | F: CACGCGGAGGTCAAGCTAC | NM_008261.3 |
|  | R: CCCAGAGATGGGAGAGGTGAT |  |
| PCNA | F: TTGCACGTATATGCCGGGACC | NM_022381.3 |
|  | R: GCTGAACTGGCTCATTCATCTCT |  |
| Skp2 | F: ATGGACTGCTCTCAAACCTCG | NM_013787.3 |
|  | R: CCTGGAAAGTTCTCCCGACTAA |  |
| TGF-β2 | F: TCGACATGGATCAGTTTATGCG | NM_009367.4 |
|  | R: CCCTGGTACTGTTGTAGATGGA |  |
| TNFα | F: CCTGTAGCCCACGTCGTAG | NM_013693.3 |
|  | R: GGGAGTAGACAAGGTACAACCC |  |
