# Supplementary material for: GITRL impairs hepatocyte repopulation by liver progenitor cells to aggravate inflammation and fibrosis by GITR+CD8+ T lymphocytes in CDE Mice
Source: Cell Death Dis. 2024 Feb 6;15(2):114. doi: 10.1038/s41419-024-06506-y (PMC10847460; doi:10.1038/s41419-024-06506-y)
Supplement: Supplementary file 3 — Supplementary figures [file 41419_2024_6506_MOESM3_ESM.docx]

**GITRL Impairs Hepatocyte Repopulation of Liver Progenitor Cells to Aggravate Inflammation and Fibrosis by GITR^+^CD8^+^ T Lymphocytes in CDE Mice**

Li Li,^1,2,3,5^ Yu He,^1,2,3,5^ Kai Liu,^4^ Lin Liu,^1,2,3^ Shan Shan,^1,2,3^ Helin Liu,^1,2,3^ Jiangbo Ren,^1,2,3^ Shujie Sun,^1,2,3^ Min Wang,^1,2,3^ Jidong Jia,^1,2,3,*^ and Ping Wang^1,2,3,^*

^1^ Liver Research Center, Beijing Friendship Hospital, Capital Medical University, Beijing 100050, China

^2^ National Clinical Research Center for Digestive Disease, Beijing 100069, China

^3^ Beijing Key Laboratory on Translational Medicine on Cirrhosis, Beijing 100050, China

^4^ Beijing Clinical Research Institute, Beijing 100050, China

^5^ These authors contributed equally: Li Li and Yu He.

*Correspondence: Ping Wang, wangping2009@ccmu.edu.cn, Jidong Jia, [jia_jd@ccmu.edu.cn](mailto:jia_jd@ccmu.edu.cn), Beijing Friendship Hospital, Capital Medical University, No. 95 Yong An Road, Beijing 100050, China. TEL/FAX: 86-10-63142315

**RESULTS：**


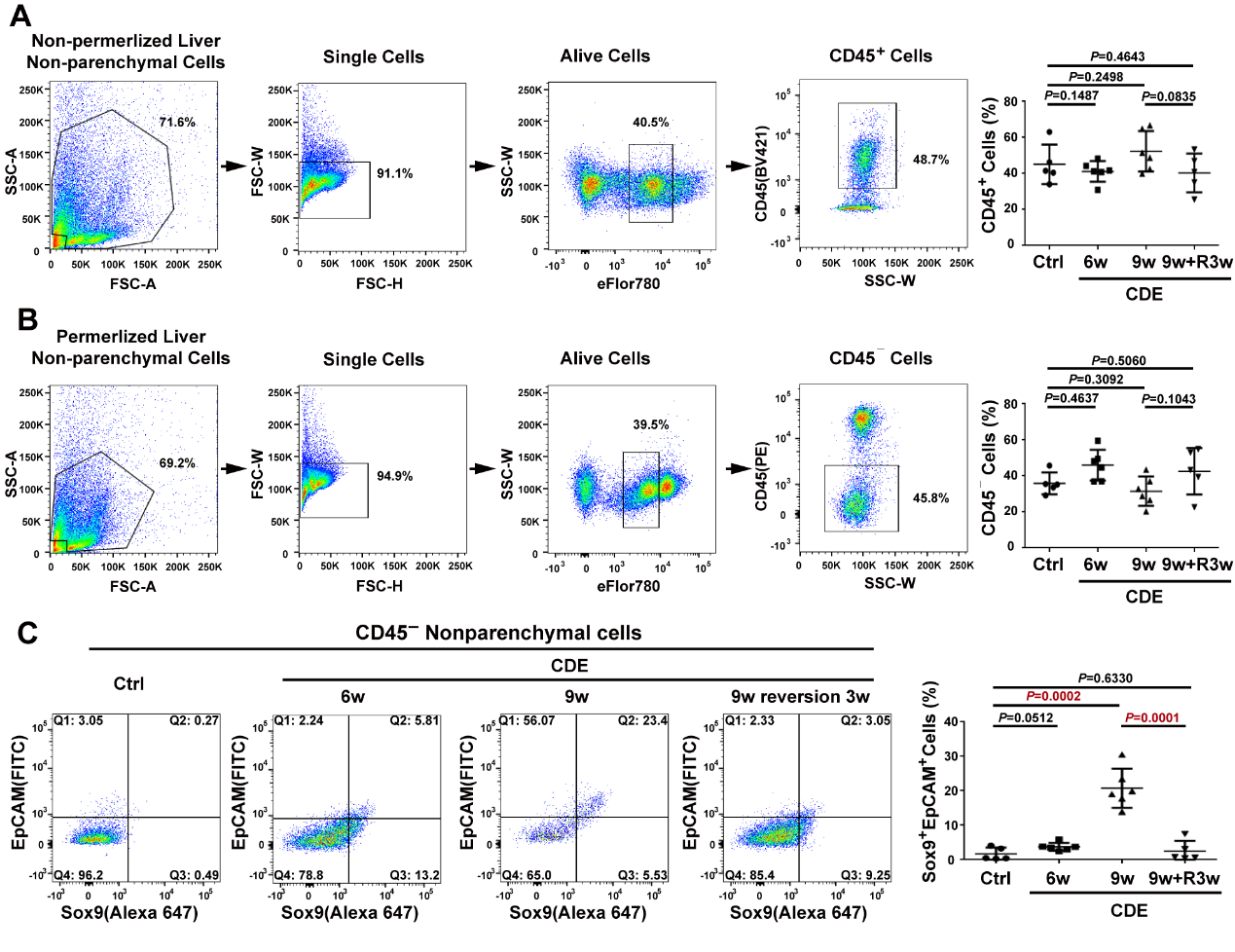


**Figure S1. The proportion of** **CD45^+^ liver infiltrating immune cells, CD45^－^liver non-parenchymal cells, and EpCAM^+^Sox9^+^ liver progenitor cells after CDE-injury and recovery. A** Representative flow cytometry images of the gating strategy to identify the non-permerlized CD45^+^ liver infiltrating immune cells and their proportion among liver non-parenchymal cells. **B** Representative flow cytometry images of the gating strategy to identify the permerlized CD45^－^ cells and their proportion among liver non-parenchymal cells. **C** Representative flow cytometry images and quantifications of the proportion of Sox9^+^EpCAM^+^ liver progenitor cells among the CD45 ^̶^ liver nonparenchymal cells.


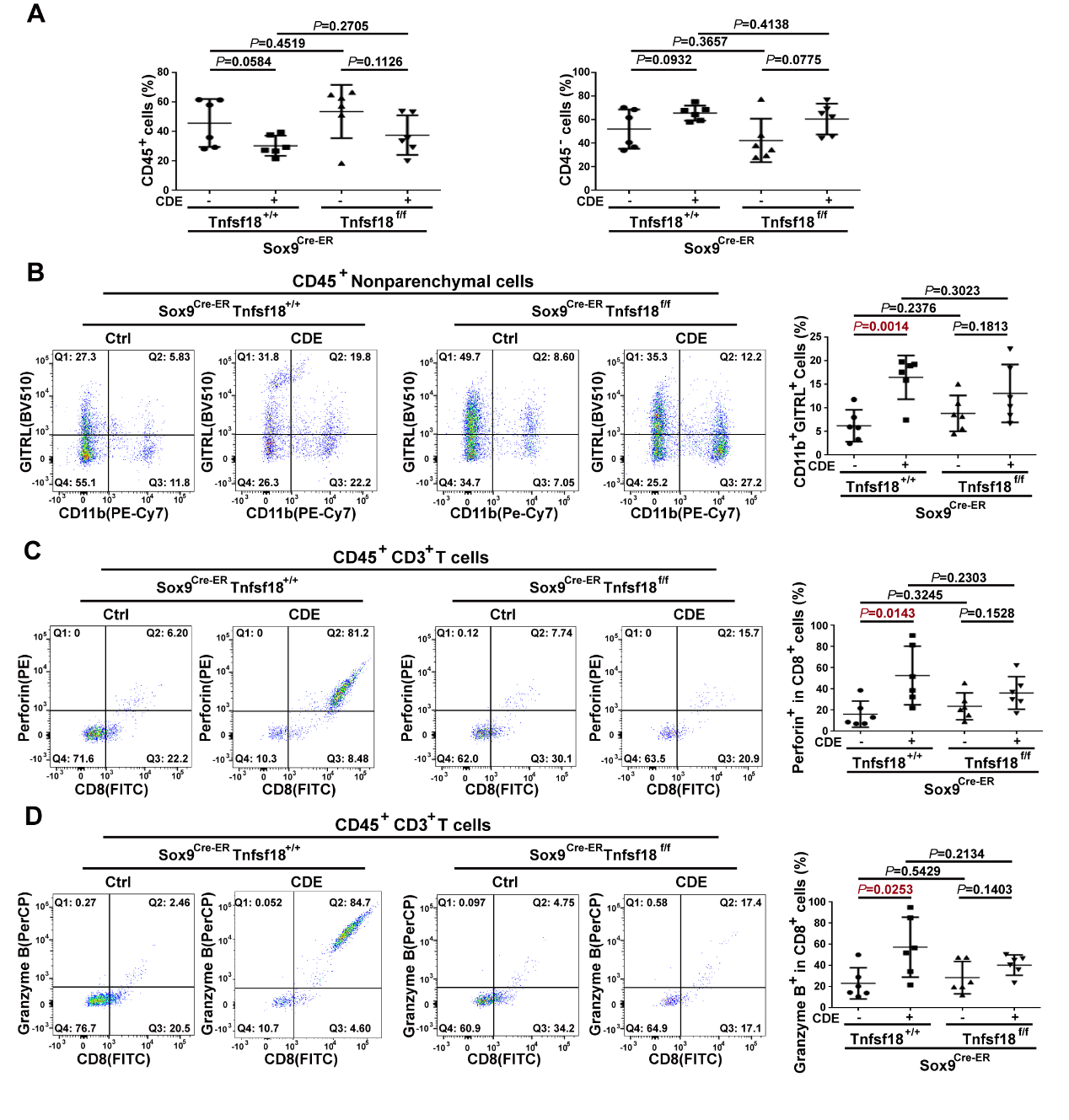


**Figure S2. The proportion of CD45^+^ liver infiltrating immune cells, CD45^－^liver non-parenchymal cells, and functional analysis of CD8^+^ T lymphocytes in CDE-injured GITRL conditional knockout mice. A** The proportion of the CD45^+^ liver infiltrating immune cells and the CD45^－^ cells among liver non-parenchymal cells. **B** Representative flow cytometry images and statistical quantification of the proportion of GITRL^+^CD11b^+^ myeloid cells among the liver infiltrating CD45^+^ immune cells. **C** Representative flow cytometry images and statistical quantification of the perforin-positive proportion of CD8 T lymphocytes. **D** Representative flow cytometry images and statistical quantification of the granzyme B-positive proportion of CD8 T lymphocytes.


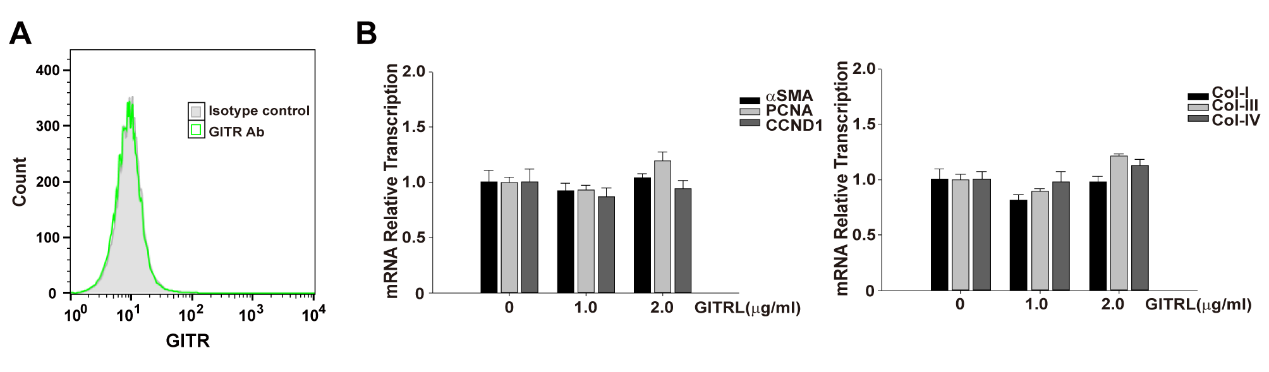


**Figure S3. Hepatic stellate cells did not express GITR and showed no response to GITRL *in vitro*.** **A** Immunofluorescence staining and flow cytometry analysis of the GITR expression in human hepatic stellate cell line LX-II. **B** Real-time PCR analysis showed neither 1μg/ml nor 2μg/ml GITRL stimulated more than 1.2 folds of αSMA, collagen I and Collagen III transcription in LX-II cells.
